# Supplementary material for: Deep learning predicts all-cause mortality from longitudinal total-body DXA imaging
Source: Commun Med (Lond). 2022 Aug 16;2:102. doi: 10.1038/s43856-022-00166-9 (PMC9381587; doi:10.1038/s43856-022-00166-9)
Supplement: Supplementary file 1 — Supplemental Material [file 43856_2022_166_MOESM1_ESM.pdf]

DEEP LEARNING PREDICTS ALL-CAUSE MORTALITY FROM LONGITUDINAL TOTAL-BODY DXA IMAGING

Yannik Glaser*[1], John Shepherd[2], Lambert Leong[2], Thomas Wolfgruber[2], Li-Yung Lui[3], Peter Sadowski[1], Steven R. Cummings[3]

[1] Information and Computer Sciences, University of Hawai'i at Mānoa, Honolulu, HI

[2] University of Hawai'i at Mānoa Cancer Center, Honolulu, HI

[3] San Francisco Coordinating Center, California Pacific Medical Center Research Institute, San Francisco, CA

SUPPLEMENTARY INFORMATION

SUPPLEMENTARY METHODS

Model hyperparameter tuning

All models were initially individually tuned using the SHERPA[1] package. For every model, a range of possible values is defined for each hyperparameter that is being tuned and then a random search is run over the entire possible hyperparameter space to obtain the best configuration. The number of runs depends on the model, a detailed breakdown of parameters, parameter ranges, and number of runs for each model is provided in the Supplementary Table 1. Afterwards, small adjustments were made manually.

Cause-of-death subgroup performance

Unlike Table 2 in the main text, primary and secondary causes of death are separated in this table, thus counts for cardiovascular disease and cancer differ from the main text. Similarly, though, the cause-of-death subgroups are still determined based only on the combination of participants who died within the follow-up window of the Health ABC study and for whom cause-of-death information was available, serving as positive examples, and participants who lived either until the study follow-up period concluded or for at least 10 years after their last available scan, serving as negative examples. While other causes of death were recorded as part of the study, their sample sizes are negligible and are thus omitted for this analysis.

	Image-only single-record	Metadata-only single-record	Combined single-record	Sequence Model
**Random search iterations**	200	100	200	100
**Batch size**	[8, 32, 64]	[16, 64, 128, 256]	[8, 32, 64]	[16, 64, 128, 256]
**Learning rate**	[0.0005, 0.001, 0.005, 0.01]	[0.0005, 0.001, 0.005, 0.01]	[0.0005, 0.001, 0.005, 0.01]	[0.0005, 0.001, 0.005, 0.01]
**Optimizer**	[Adam, RAdam, Ranger, SGD]	[Adam, SGD]	[Adam, RAdam, Ranger, SGD]	[Adam, SGD]
**Architecture**	[DenseNet121, NASNet, Inceptionv2]	Not included	Not included	Not included
**Model depth**	Not included	1 - 8	1 - 10 (After concatenation)	1 - 10 (After LSTM layer)
**Dense layer size**	Not included	8 - 100	[128, 256, 512, 1048, 2048]	[8, 16, 32, 64, 128]
**Dropout rate (final layer)**	[0.0, 0.5]	[0.0, 0.5]	[0.0, 0.5]	[0.0, 0.5]
**Epochs**	20 - 50	20 - 200	Not included	20 - 1000
**Image augmentation probability**	0.0 - 0.5	Not included	Not included	Not included
**Early stopping patience**	[5, 10, 15]	[5, 10, 30, 50]	[5, 10, 15]	[5, 10, 30, 50]
**Weight initialization**	[Imagenet, Glorot uniform]	Not included	Not included	Not included
**Record dropout probability**	Not included	Not included	Not included	0.0 - 0.5

Supplementary Table 1. Hyperparameter ranges used during hyperparameter tuning with SHERPA. Hyphens indicate any value within the specified range, brackets indicate a choice between the listed options. “Not included” indicates that this parameter was either hand-tuned or does not apply to this architecture.

	Black		White		Female		Male		CVD		Cancer	
	Baseline (n=258)	Most recent (n=258)	Baseline (n=381)	Most recent (n=381)	Baseline (n=319)	Most recent (n=319)	Baseline (n=320)	Most recent (n=320)	Baseline (n=360)	Most recent (n=360)	Baseline (n=333)	Most recent (n=333)
**Image model**	0.60 (0.52-0.67)	0.60 (0.53-0.68)	0.67 (0.61-0.72)	0.66 (0.61-0.72)	0.58 (0.51-0.64)	0.59 (0.52-0.65)	0.62 (0.56-0.69)	0.62 (0.55-0.69)	0.71 (0.64-0.77)	0.69 (0.63-0.74)	0.64 (0.56-0.72)	0.65 (0.59-0.71)
**Metadata model**	0.68 (0.61-0.74)	0.69 (0.64-0.76)	0.66 (0.60-0.72)	0.75 (0.70-0.80)	0.66 (0.59-0.72)	0.70 (0.64-0.76)	0.65 (0.59-0.71)	0.73 (0.67-0.79)	0.73 (0.67-0.79)	0.76 (0.72-0.82)	0.70 (0.64-0.77)	0.70 (0.63-0.73)
**Combined-modality model**	0.68 (0.62-0.75)	0.70 (0.64-0.77)	0.67 (0.61-0.73)	0.77 (0.72-0.82)	0.67 (0.60-0.73)	0.70 (0.64-0.76)	0.68 (0.61-0.74)	0.76 (0.70-0.81)	0.76 (0.70-0.81)	0.78 (0.72-0.82)	0.71 (0.64-0.78)	0.71 (0.63-0.76)

Supplementary Table 2. Single-record model area under the receiver operating characteristic (AUROC) scores and 95% confidence intervals for full test set (“overall”) and only the most recent scan for each participant in the test set.

	Primary cause of death				Underlying Cause of death			
	Cardiovascular (n = 475)	Sepsis (n = 126)	Respiratory Failure (n = 78)	Pneumonia (n = 78)	Cardiovascular (n = 548)	Cancer (n = 452)	Dementia (n = 193)	COPD (n = 64)
**Image Model**	0.64 (0.61-0.67)	0.71 (0.67-0.76)	0.62 (0.55-0.68)	0.77 (0.70-0.82)	0.65 (0.62-0.67)	0.66 (0.63-0.69)	0.55 (0.51-0.60)	0.68 (0.60-0.76)
**Metadata model**	0.73 (0.70-0.75)	0.74 (0.70-0.79)	0.66 (0.59-0.73)	0.77 (0.71-0.82)	0.74 (0.72-0.76)	0.69 (0.66-0.71)	0.68 (0.63-0.72)	0.80 (0.73-0.87)
**Combined -modality model**	0.73 (0.71-0.76)	0.76 (0.72-0.81)	0.65 (0.58-0.72)	0.81 (0.75-0.85)	0.75 (0.73-0.77)	0.70 (0.67-0.73)	0.70 (0.66-0.75)	0.84 (0.77-0.90)
**Image sequence**	0.74 (0.68-0.79)	0.71 (0.61-0.81)	0.72 (0.58-0.86)	0.80 (0.69-0.90)	0.72 (0.67-0.78)	0.72 (0.66-0.78)	0.68 (0.60-0.76)	0.85 (0.72-0.95)
**Metadata sequence**	0.80 (0.74-0.84)	0.81 (0.72-0.88)	0.76 (0.62-0.87)	0.76 (0.63-0.87)	0.80 (0.75-0.84)	0.73 (0.66-0.79)	0.74 (0.67-0.82)	0.83 (0.71-0.92)
**Combined -modality sequence**	0.81 (0.76-0.86)	0.82 (0.74-0.89)	0.78 (0.66-0.88)	0.77 (0.65-0.88)	0.81 (0.77-0.86)	0.78 (0.72-0.83)	0.78 (0.71-0.85)	0.88 (0.80-0.95)

Supplementary Table 3. Cause-of-death performance analysis for four most common primary and underlying causes of death in the Health ABC dataset. Scores show area under the receiver operating characteristic (AUROC) and 95% confidence intervals.

	**Baseline Diabetes Status**			**Baseline BMI Group**			
	Normal (n = 2171)	Prediabetic (n = 1036)	Diabetic (n = 198)	Underweight (n = 59)	Normal (n = 1199)	Overweight (n = 1603)	Obese (n = 859)
**Image Model**	0.65 (0.62-0.67)	0.58 (0.55-0.67)	0.65 (0.57-0.72)	0.74 (0.59-0.86)	0.67 (0.64-0.71)	0.65 (0.62-0.67)	0.54 (0.50-0.58)
**Metadata model**	0.69 (0.64-0.74)	0.67 (0.64-0.70)	0.71 (0.64-0.78)	0.85 (0.75-0.94)	0.72 (0.69-0.75)	0.70 (0.67-0.73)	0.64 (0.60-0.76)
**Combined-modality model**	0.71 (0.69-0.73)	0.70 (0.66-0.73)	0.73 (0.65-0.80)	0.85 (0.73-0.94)	0.85 (0.73-0.94)	0.72 (0.70-0.75)	0.65 (0.61-0.69)
**Image sequence**	0.70 (0.66-0.75	0.70 (0.64-0.76)	0.78 (0.65-0.89)	0.73 (0.46-0.95)	0.74 (0.68-0.80)	0.73 (0.67-0.79)	0.62 (0.54-0.70)
**Metadata sequence**	0.73 (0.69-0.78)	0.73 (0.67-0.78)	0.76 (0.64-0.86)	0.83 (0.57-0.98)	0.74 (0.68-0.79)	0.77 (0.72-0.82)	0.70 (0.62-0.77)
**Combined-modality sequence**	0.77 (0.72-0.81)	0.77 (0.72-0.83)	0.84 (0.73-0.92)	0.85 (0.62-0.99)	0.76 (0.71-0.82)	0.80 (0.75-0.84)	0.75 (0.68-0.82)

Supplementary Table 4. Additional subgroup performance analysis for all models on diabetes-status and BMI-category subgroups. Scores show area under the receiver operating characteristic (AUROC) and 95% confidence intervals. Only relatively small sample sizes were available for diabetic and underweight subgroups.

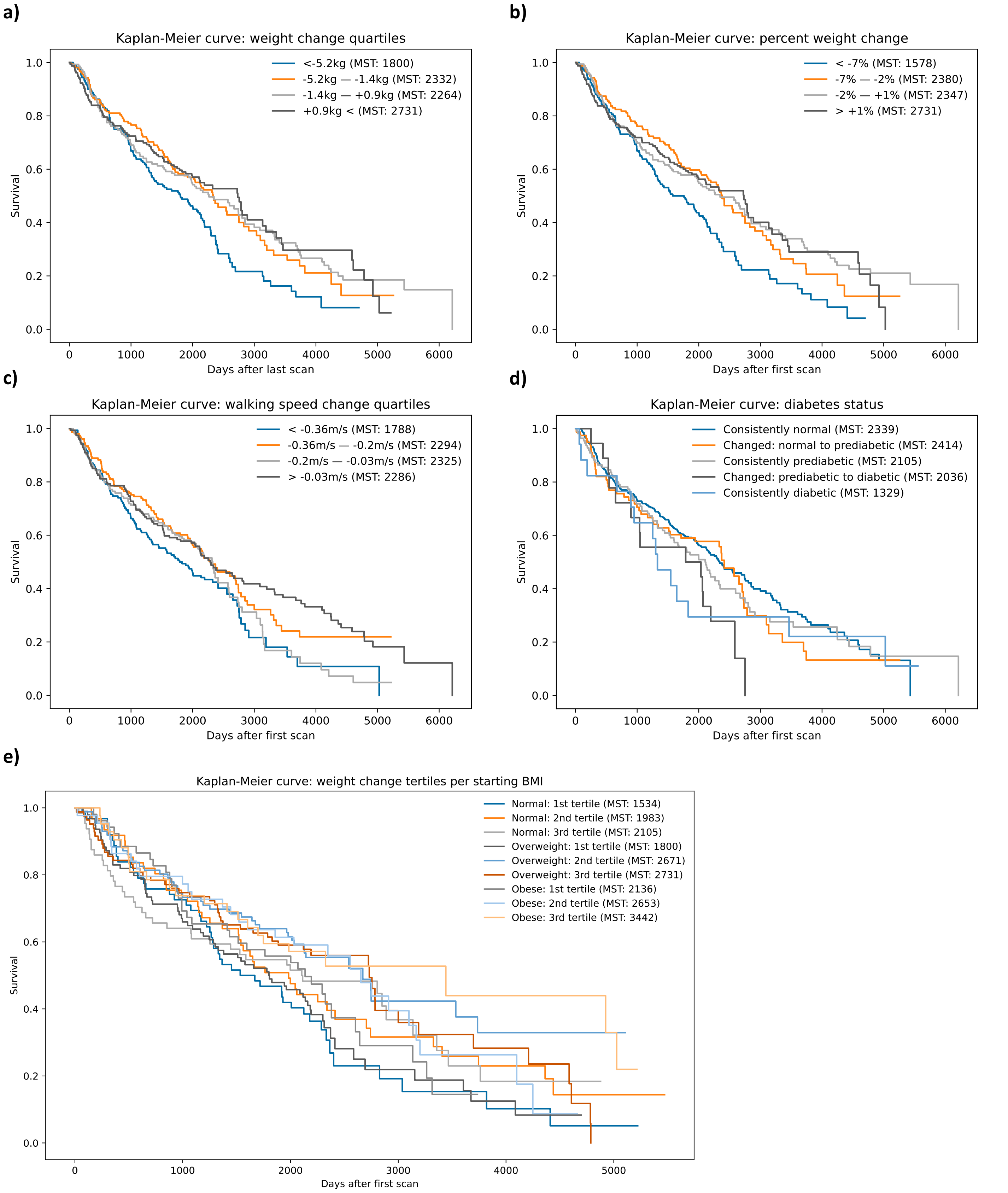

Supplementary Figure 1. Kaplan-Meier curves and median survival time (MST) on different subsets of the test data. **a,** Survival curves for participant quartiles of absolute weight change between first scan and death. **b,** Survival curves for participant quartiles of percent weight change between first scan and death. **c,** Survival curves for participant quartiles of absolute change in walking speed between first scan and death. **d,** Survival curves for participants based on evolution of diabetes status between first scan and death. Diabetes status is calculated based on fasted blood glucose with standard clinical cutoffs. **e,** Survival curves for participant tertiles based on absolute weight change between first scan and death, further stratified by initial BMI classification using standard clinical cutoffs.

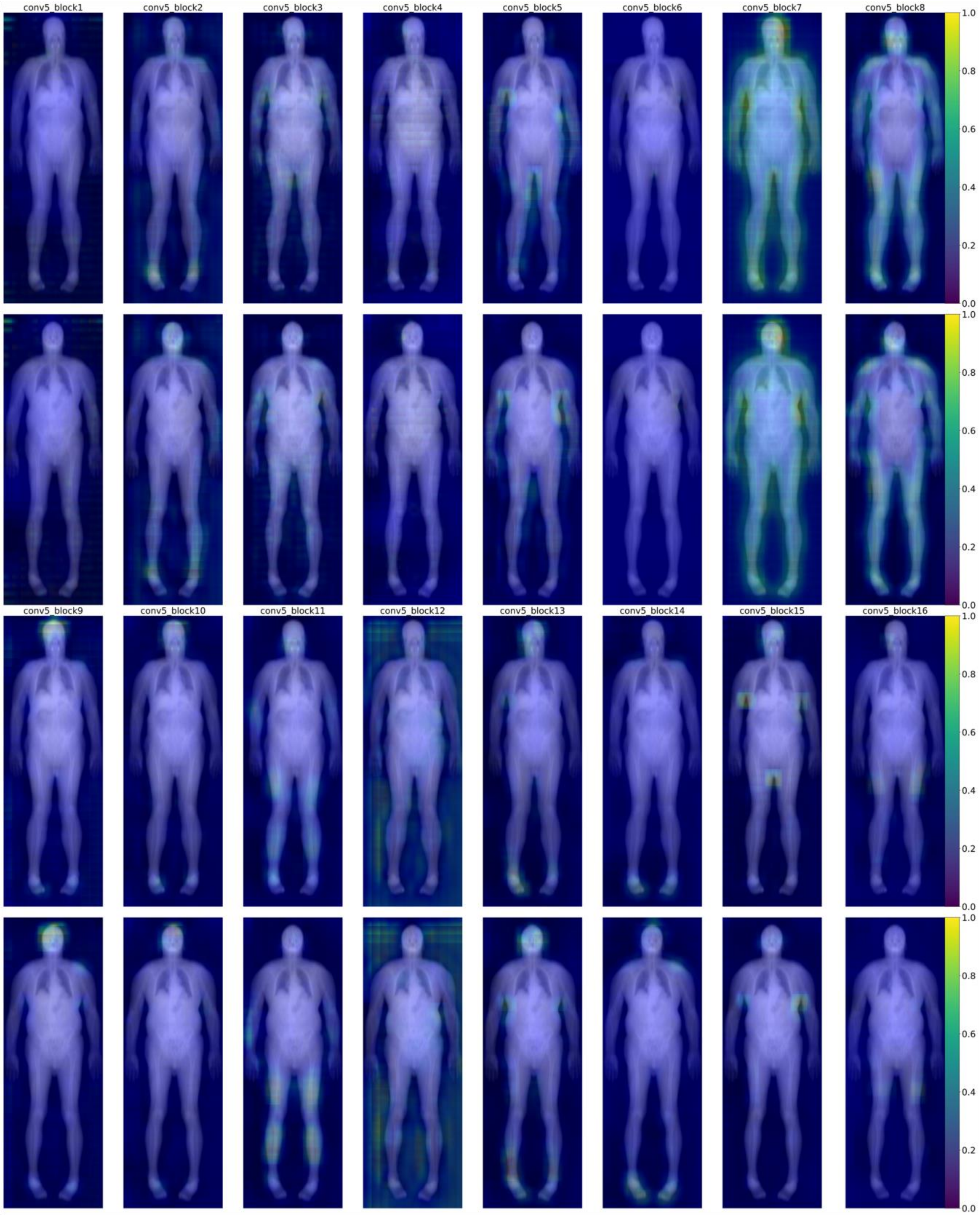

Supplementary Figure 2. Full Grad-CAM feature maps for all convolutional layers within the last dense block of the combined-modality single-record model.

SUPPLEMENTARY REFERENCES

1. Hertel, L., Collado, J., Sadowski, P., Ott, J. & Baldi, P. Sherpa: Robust hyperparameter optimization for machine learning. *SoftwareX* **12**, 100591 (2020).
