# Supplementary material for: Deep learning predicts all-cause mortality from longitudinal total-body DXA imaging
Source: Commun Med (Lond). 2022 Aug 16;2:102. doi: 10.1038/s43856-022-00166-9 (PMC9381587; doi:10.1038/s43856-022-00166-9)
Supplement: Supplementary file 8 — Description of Additional Supplementary Files [file 43856_2022_166_MOESM8_ESM.pdf]

Description of Additional Supplementary Files

File Name: Supplementary Data 1

Description: Supplementary data to reproduce Figure 2

File Name: Supplementary Data 2

Description: Supplementary data to reproduce Figure 3a

File Name: Supplementary Data 3

Description: Supplementary data to reproduce Figure 3b

File Name: Supplementary Data 4

Description: Supplementary data to reproduce Figure 4a

File Name: Supplementary Data 5

Description: Supplementary data to reproduce Figure 4b
